# Artificial intelligence-based morphometric signature to identify ductal carcinoma in situ with low risk of progression to invasive breast cancer

**DOI:** 10.21203/rs.3.rs-3639521/v1

**Published:** 2023-12-13

**Authors:** Marcelo Sobral-Leite, Simon Castillo, Shiva Vonk, Xenia Melillo, Noomie Lam, Brandi de Bruijn, Yeman Hagos, Joyce Sanders, Mathilde Almekinders, Lindy Visser, Emma Groen, Petra Kristel, Caner Ercan, Leyla Azarang, Yinyin Yuan, Grand Challenge PRECISION Consortium, Renee Menezes, Esther Lips, Jelle Wesseling

**Affiliations:** Netherlands Cancer Institute; The University of Texas MD Anderson Cancer Center; Netherlands Cancer Institute; Netherlands Cancer Institute; Netherlands Cancer Institute; Netherlands Cancer Institute; The Institute of Cancer Research; Netherlands Cancer Institute; Netherlands Cancer Institute; Netherlands Cancer Institute; Netherlands Cancer Institute; Netherlands Cancer Institute; The University of Texas MD Anderson Cancer Center; Netherlands Cancer Institute; The Institute of Cancer Research, London; Netherlands Cancer Institute; Netherlands Cancer Institute; Netherlands Cancer Institute; Netherlands Cancer Institute

**Keywords:** DCIS, biomarkers, digital pathology, artificial intelligence

## Abstract

Ductal carcinoma in situ (DCIS) may progress to ipsilateral invasive breast cancer (iIBC), but often never will. Because DCIS is treated as early breast cancer, many women with harmless DCIS face overtreatment. To identify these women that may forego treatment, we hypothesized that DCIS morphometric features relate to the risk of subsequent iIBC.

We developed an artificial intelligence-based DCIS morphometric analysis pipeline (AIDmap) to detect DCIS as a pathologist and measure morphological structures in hematoxylin-eosin-stained (H&E) tissue sections. These were from a case-control study of patients diagnosed with primary DCIS, treated by breast-conserving surgery without radiotherapy. We analyzed 689 WSIs of DCIS of which 226 were diagnosed with subsequent iIBC (cases) and 463 were not (controls). The distribution of 15 duct morphological measurements in each H&E was summarized in 55 morphometric variables. A ridge regression classifier with cross validation predicted 5-years-free of iIBC with an area-under the curve of 0.65 (95% CI 0.55–0.76). A morphometric signature based on the 30 variables most associated with outcome, identified lesions containing small-sized ducts, low number of cells and low DCIS/stroma area ratio. This signature was associated with lower iIBC risk in a multivariate regression model including grade, ER, HER2 and COX-2 expression (HR = 0.56; 95% CI 0.28–0.78). AIDmap has potential to identify harmless DCIS that may not need treatment.

## Introduction

Ductal carcinoma in situ (DCIS) may progress to invasive breast cancer (IBC), but 3 out of 4 patients never will if left untreated (1, 2, 3). Since the introduction of population-based breast cancer screening, the incidence of DCIS has increased at least seven-fold (4). In current practice, we are not able to distinguish the minority of DCIS that is prone to progress to IBC from those that never will (5, 6). Due to this uncertainty, almost all women with DCIS are treated with surgery, often followed by radiotherapy (7). This implies that many women with harmless DCIS carry the burden of intensive treatment without any benefit (8, 9). Therefore, there is an urgent need to classify DCIS lesions that will remain indolent, and those that might progress to IBC to prevent overtreatment of low-risk DCIS.

Several studies explored classical morphological and molecular features to predict ipsilateral subsequent IBC (iIBC) after DCIS (10). Although DCIS grade, expression of the estrogen receptor (ER), human epidermal growth factor receptor 2 (HER2) and prostaglandin-endoperoxide synthase 2 (COX-2) have shown an association with invasive progression (10, 11, 12, 13), the clinical utility of their prognostic value is a subject of ongoing debate (9, 10, 11, 12, 13, 14, 15, 16, 17).

Conflicting results are published on the relationship between DCIS lesion size and risk of progression to iIBC, may be because assessment of DCIS size on macroscopic examination of a specimen is not highly accurate (18, 19, 20). At the microscopy level, it is also notoriously difficult to estimate the extent of DCIS accurately after tissue has been sectioned, also because a standardized method measuring the DCIS lesion size is lacking (19, 21). In addition, there is also an obvious knowledge gap on the prognostic value of morphological measurements in hematoxylin-eosin-stained (H&E) slides (22, 23, 24, 25). To address this, we developed an artificial intelligence-based DCIS Morphometric Analysis Pipeline (AIDmap) to detect, measure and quantify DCIS features with high accuracy and reproducibility. Measurements were obtained from scanned whole-slide images (WSIs) of H&E sections from primary DCIS lesions of a large retrospective study in The Netherlands (13, 26).

We hypothesized that objective, reproducible, and accurate measurements of morphometric features of DCIS lesions using AIDmap could help to stratify risk of progression to IBC. Ultimately, this may aid DCIS management decisions. First, by sparing many women with low-risk DCIS the burden of potential overtreatment. Second, by adequate treatment of women with high-risk DCIS, thereby not compromising the excellent outcomes of DCIS management currently achieved.

## Results

### AIDmap development

**Figure 1:**
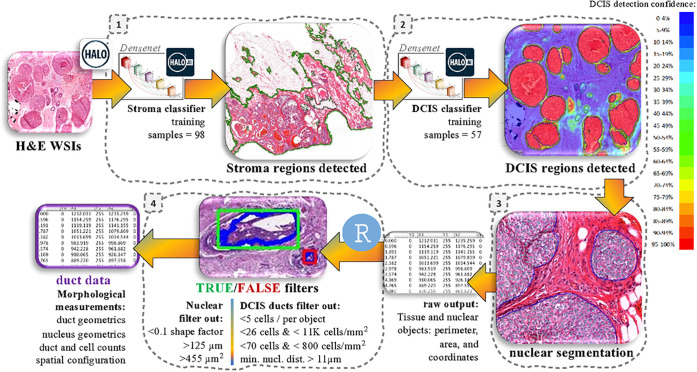
AIDmap workflow. HALO deep learning neural network was trained to recognize morphological structures in H&E whole-slides images (WSIs). **1:** The first classifier was trained to annotate the fibroglandular tissue (stroma), leaving adipocytes outside (green line). **2:** DCIS classification was applied within the annotated stroma, by detecting pixels that reached more than 90% of confidence of composing a DCIS duct (red areas in the image heatmap). **3:** Next, a nuclear segmentation sensing hematoxylin staining was applied within the DCIS regions to detect all nuclear structures (supplementary appendix section S1.1). After these three steps, HALO provided tables containing the area, perimeter and spatial coordinates of stroma, DCIS and nuclear objects that were imported to R studio. **4:** A True/False computational filtering was applied according to the nuclear perimeter, area and circular shape factor in order to eliminate false nuclear objects. And a True/False filtering was applied on DCIS objects, according the density of cells and average minimal nuclear distance (min. nucl. dist.) within the duct, to eliminate false DCIS ducts detected by HALO (supplementary appendix section S1.2). Finally, morphological measurements for each DCIS duct were obtained (supplementary appendix section S1.4).

We obtained H&E WSIs from a nested case-control study of patients diagnosed with primary DCIS, treated by breast-conserving surgery without radiotherapy (methods). Patients diagnosed with iIBC during follow-up were considered as “cases” and those with no invasive diagnosis considered as “controls” (13). Pathologists digitally annotated stroma and DCIS regions in H&E WSIs (supplementary appendix, section S1.1). These annotations were used by the HALO AI module, a deep learning neural network that created a trained-by-example tissue classifiers to detect stroma areas and DCIS ducts. Additionally, we applied a nuclei segmentation to detect the nucleus of cells within the ducts. HALO platform estimated the area, the perimeter and the spatial coordinates of these stroma areas, DCIS ducts and cell nucleus (figure 1). Finally, we applied a computational filtering to improve the detection accuracy of DCIS ducts (figure 1, methods and supplementary appendix section S1.2). This resulted in our artificial intelligence-based DCIS morphometric analysis pipeline (AIDmap).

### Technical validation of DCIS segmentation

To validate the detection accuracy of the DCIS segmentation used in the AIDmap, we applied it on 20 H&E sections of DCIS lesions from an independent cohort (Translational Breast Cancer Research Consortium, TBCRC) (27). All DCIS lesions within these 20 H&E slides were annotated by a pathologist, blinded to the AIDmap detection. The validation consisted of quantifying the spatial overlap of the computational prediction and the pathologist’s annotations using the intersection-over-union (IOU) score (supplementary figure 1A-C) (28). Spatial overlap between computational prediction and pathologist’s annotation had a median IOU score of 0.76 (interquartile range = 0.68 – 0.83; supplementary figure 1D). Additionally, we compared the number of DCIS ducts detected by the pathologist and by AIDmap using Pearson’s correlation coefficient. The number of DCIS lesions assigned by the pathologist was strongly and significantly correlated with the number predicted by AIDmap: r = 0.79 (95% CI 0.44 – 0.89, p = 2.9×10^−5^; supplementary figure 1E).

### Building a DCIS morphometrics classifier

**Figure 2:**
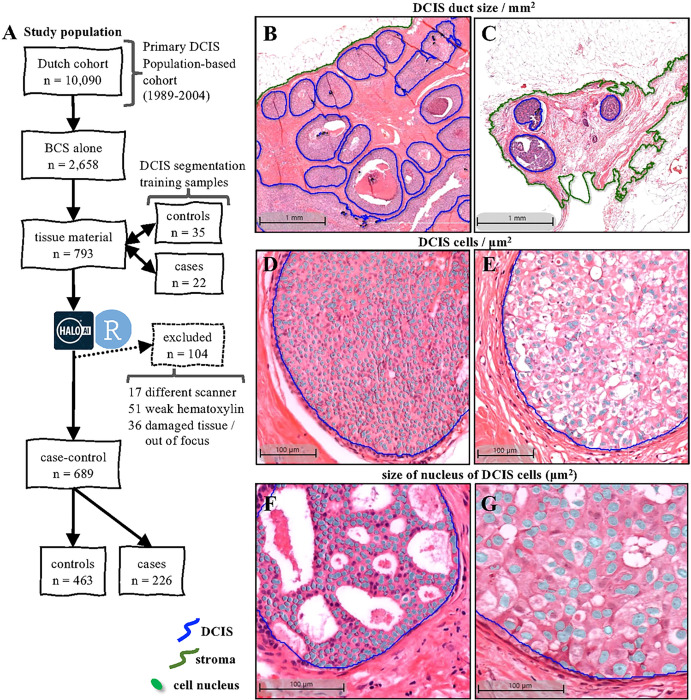
Sample and classification details. **A)** Flow chart of the study population of patients diagnosed with primary DCIS in the Netherlands between 1989 and 2004. For training in the HALO AI module, 57 H&E sections from DCIS treated with BCS alone were used for automated stroma and DCIS segmentation. In total, 689 H&E WSI were successfully analyzed and their images revealed the variability on the density of DCIS ducts: large **(B)** and small **(C)** duct size (mm2); density of DCIS cells within the ducts: high **(D)** and low **(E)** DCIS cells/μm^2^; and average size of DCIS nucleus of the cells within the DCIS ducts: **(F)** large and small **(G)** DCIS nucleus (μm2); among other morphometric variables that varied among the samples.

In total, we uploaded 793 H&E WSIs of primary DCIS lesions from 793 patients treated with BCS only. However, after visual inspection, 104 did not achieve the minimal quality for computational segmentation due to weak hematoxylin or eosin staining, tissue section damage, artefacts, out-of-focus regions, or images scanned with scanner device out of the standard (figure 2A). We successfully applied AIDmap on 689 WSIs, in which 463 were assigned as controls and 226 assigned as cases (figure 2A and table 1). We detected a total of 37,020 DCIS ducts in this dataset, with a median of 36 DCIS ducts per slide, ranging between 1 to 623.

The area, perimeter and spatial coordinates of DCIS objects, stroma and cells were the basic measurements used to calculate all possible and reasonable geometric and spatial features for each DCIS duct. In summary, we obtained 15 morphological measurements (supplementary table 1).

DCIS duct morphological measurements displayed large intra- and inter-patient variability in the whole dataset of 689 WSIs (supplementary figures 2–3). This variability, such as the area of DCIS ducts, the density of DCIS cells inside the ducts and the size of cell’s nucleus are examples of morphological structures in which the heterogeneity can also be observed by conventional microscopic examination of DCIS WSIs (figures 2B–G). AIDmap measured several of these morphological structures and their spatial arrangement observed in WSIs with high accuracy and precision.

### Assessing the prognostic value of morphometric features

**Figure 3:**
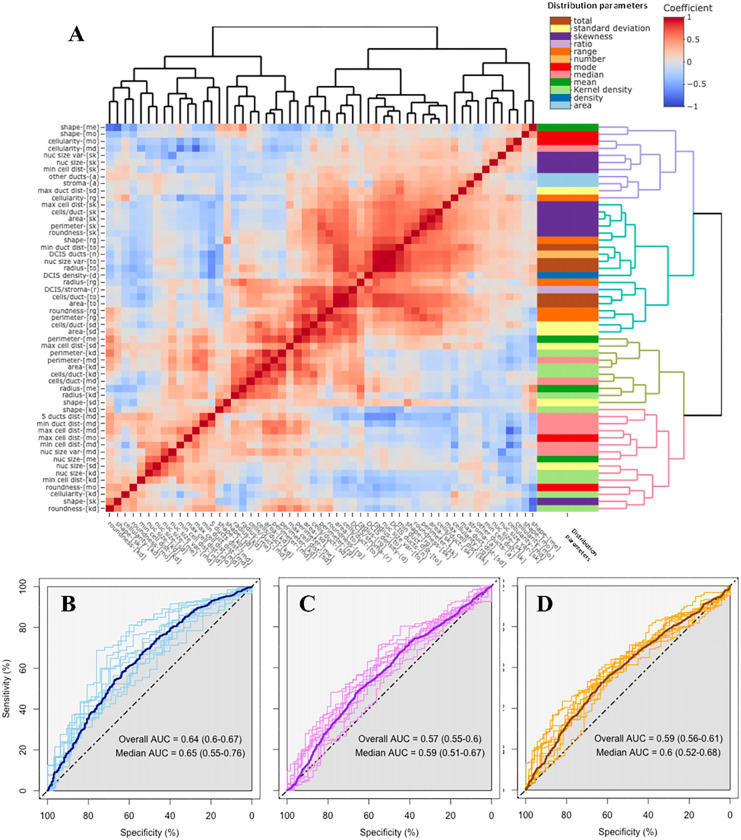
Analysis of the morphometric variables. **A)** Heatmap with the Spearman’s rank correlation coefficients between the 55 variables obtained from the AIDmap in each H&E slide. Row side colors represent the parameters used to calculate each morphometric variable. Abbreviations are listed in supplementary appendix (section S1.4). Receiver operating characteristic (ROC) curves and area under the curve (AUC) calculations from the generalized linear models to predict absence of iIBC event during follow up after 5, 10 or 15 years (**B-D**, respectively).

We further summarized the values of these morphological measurements in each WSI with the aim to reproduce the morphometric inter and intra-variability (supplementary appendix section S1.4). In brief, we calculated 8 parameters of the distribution of the 15 morphological measurements in each WSI (supplementary figure 4A), yielding in total 120 variables. Of these, 55 non-redundant variables were selected with representative potential to describe the morphometric heterogeneity of DCIS lesions (supplementary table 2 and supplementary figure 4B). These 55 morphometric variables were mostly weakly correlated (85% had Spearman’s correlation coefficient between −0.5 and 0.5; see figure 3A).

To assess the performance of the DCIS morphometric variables to predict 5, 10, and 15 years free of iIBC progression after primary DCIS diagnosis, we built a classifier using a logistic-ridge regression based upon these 55 morphometric variables. After 10-fold double-loop cross validation, we evaluated the prediction accuracy in the test set from each loop. We obtained a median AUC of 0.65 (95% CI 0.55 – 0.78) to predict 5-years free of subsequent iIBC, 0.59 (95% CI 0.51 – 0.67) to predict 10-years and 0.60 (95% CI 0.52 – 0.68) to predict 15-years (figure 3B–D).

### Constructing a morphometric signature

**Figure 4:**
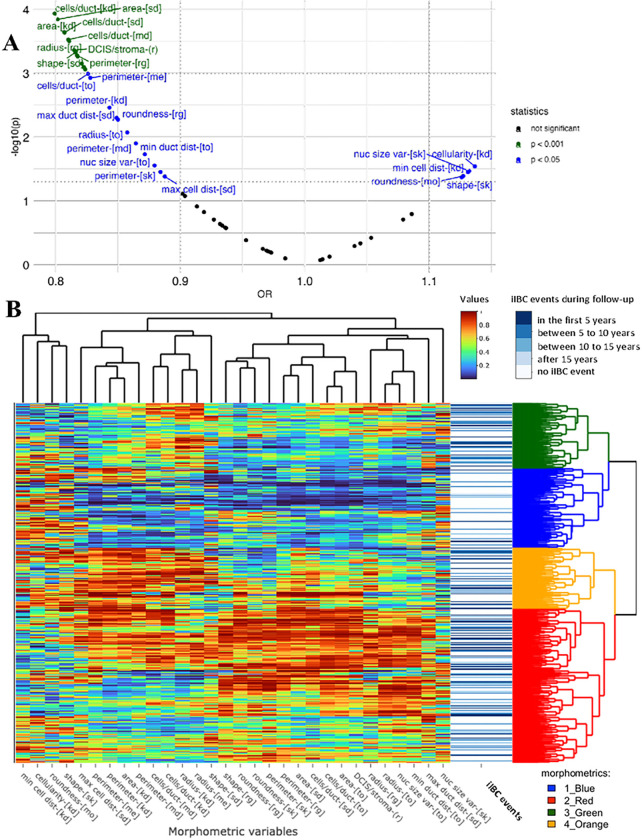
Morphometric signature of DCIS. **A)** Volcano plot showing the odds ratios (OR) and the p values (p) of the 55 morphometric variables, obtained from linear regression analysis according the iIBC status during follow-up. **B)** Heatmap of the hierarchical cluster analysis of the 30 morphometric variables statistically associated in the volcano plot. Row side colors in blue degrees represent the categories of iIBC events during follow-up. The dendrogram colors highlights the 4 groups sharing morphometric similarities.

We applied univariate regression models on all the 55 morphometric variables to estimate their association with iIBC event status during follow-up in 5 levels: iIBC event diagnosed during the first 5 years (n = 83), between the 5^th^ and 10^th^ year (n = 90), between the 10^th^ and 15^th^ year (n = 33), later than the 15^th^ year of follow-up after primary DCIS diagnosis (n = 7) or no iIBC event during follow-up (n = 476). We identified 30 variables significantly associated with iIBC status (figure 4A and table 2).

**Figure 5:**
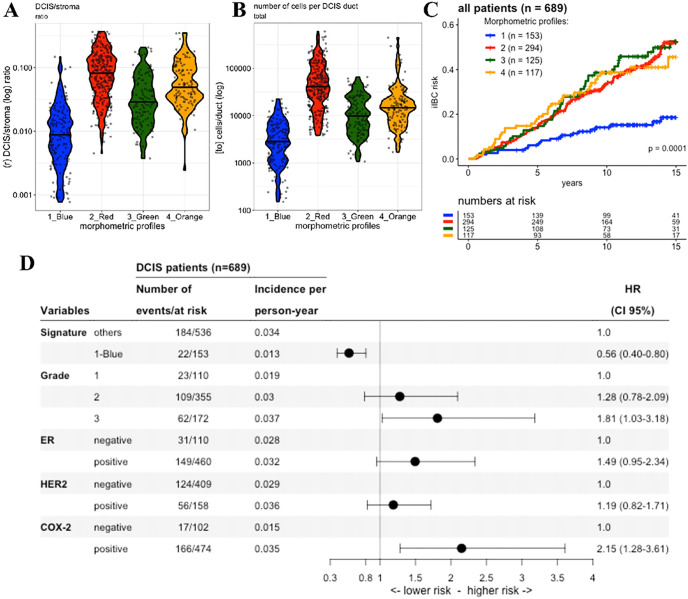
Characteristics of the 4 morphometric signatures. Differences are illustrated in the violin plots of the distribution of 4 morphometric variables among the morphometric signatures: DCIS/stroma area ratio **(A)** and the total number of cells inside DCIS ducts **(B)**. The iIBC risk curve for the patients classified with one of the morphometric signatures **(C)**, and the forest plots from the Cox multivariate regression models estimating the risk of iIBC progression during follow-up **(D).**

Subsequently, hierarchical clustering of samples using these 30 morphometric variables identified four distinct morphometric signatures (figure 4B). One clear signature (1-blue) contained lesions with significant lower average levels of total DCIS area and DCIS/stroma ratio and lower number of cells in DCIS ducts, when compared to the other morphometric signatures: 2-red, 3-green and 4-orange (all p < 0.001; figure 5A–C). In addition, the 1-blue signature showed a higher proportion of clinging/FEA growth pattern, compared with the others (supplementary figure 5A), as well as higher proportion of grade 1 DCIS (supplementary figure 5B). Other differences between signature 1-blue and the other signatures are illustrated in supplementary figures 5–7.

### Using a morphometric profile to identify low-risk DCIS lesions

By analyzing the 15 years iIBC cumulative risk curve, we observed that patients with lesions classified within the 1-Blue signature (containing lesions with small duct sizes, reduced number of cells, and lower DCIS/stroma ratio) had a significant favorable iIBC-free survival compared with the other signatures (p = 0.0001; figure 5C). The association with low-risk of iIBC events remained significant after multivariate Cox regression analysis including histopathological grade, ER, HER2 and COX-2 expression: hazard ratio (HR) = 0.56 (0.40–0.80 95%CI) (figure 5D). The 1-Blue signature also showed better iIBC-free survival among patients with DCIS lesions grade 1 or 2 (p = 0.014; supplementary figure 8A), even after the multivariate Cox regression analysis including the same features: HR= 0.58 (95%CI 0.38 – 0.88) (supplementary figure 8B).

## Discussion

To the best of our knowledge, this is the first study indicating that automatically and objectively assessed microscopic morphometry of DCIS ducts in H&E whole slide images (WSIs) relate to the risk of progression of DCIS to subsequent ipsilateral invasive breast cancer (iIBC). This was done by developing, testing, and validating our AI-based DCIS Morphometric Analysis Pipeline (AIDmap). The main features related to low-risk DCIS were smaller ducts and a lower number of DCIS cells per duct, whereas those with larger ducts and a higher cellularity are associated with a higher risk to progress to iIBC. This novel tool has the potential to identify low-risk DCIS lesions that do not need surgical intervention and/or radiotherapy, saving many women the burden of such intensive treatment.

Using AIDmap, we achieved a high degree of agreement in DCIS area detection (IOU = 0.76) and high concordance in the count of DCIS ducts compared to the annotations of the breast pathologist (r = 0.79). Importantly, we could successfully validate the accuracy and reproducibility of AIDmap in H&E WSIs of primary DCIS lesions from patients treated in different hospitals, in a different continent, stained locally in their laboratory and scanned with a different scanner than used in our department. This is highly relevant, as interobserver variability among breast pathologists is high when evaluating parameters that are based on morphological differences, such as grade and growth pattern (15, 17).

AI-tools have been developed in cancer pathology to make predictions beyond the subjective interpretation by the pathologist, including outcome and treatment response (29, 30, 31, 32). However, AI algorithms often lack interpretability regarding the predicting features that are evaluated and recognized (33). For instance, Klimov et al. (32) developed a machine learning-based model to predict recurrence risk in primary DCIS using H&E WSIs (32). They reported promising accuracy values to identify high-risk DCIS (HR = 6.39, 95%CI 3.0–13.8). However, their model was built based on features that cannot be explained or related to our current clinical and biological knowledge on DCIS. This might hamper acceptance in the clinic as a risk stratification tool. Additionally, the number of DCIS samples analyzed was limited, the patients were not uniformly treated, and the test dataset was composed by grade 3 DCIS lesions only (32).

In previous decades, the measurement of geometric features and spatial arrangement of DCIS lesions would not be feasible using classical microscopy and H&E glass slides only. Here, we developed a hand-crafted pathologist-trained AI-based tool that provides an output of explainable features relating to DCIS histology, which consequently contributes to developing trust and transparency into an AI decision making process (34). We also used a large patient dataset (n = 689), treated with BCS only, and including DCIS lesions from various histologic grade and growth patterns. It is important to mention that the use of ground truth information to train the AI algorithm can be time consuming and might feed human bias into the models (35). Since the calculation of the morphometric features and the segmentation learning process were blinded to the clinical outcome variables, AIDmap has a relatively simple architecture and lower computation cost (35).

To develop AIDmap, we have summarized the distribution parameters of the morphological measurements of DCIS ducts in H&E WSIs to reflect the inter- and intra-variability of their morphology. The morphometric signature developed in this study can be interpreted based on the results from a recent study using patient-derived mouse intraductal DCIS models, that reflected the full spectrum of DCIS morphology observed in patients (36). Hutten et al. observed that DCIS lesions more prone to progress to invasive breast cancer had larger volume and more spherical morphology (called as expansive growth pattern); when compared with DCIS lesions with lower rate of invasive progression, which had a smaller volume and more elongated shape (replacement growth pattern) (36). In the present study, as we hypothesized, lesions containing smaller DCIS areas, lower number of cells, lower DCIS stroma/ratio and less spherical ducts grouped together in the cluster analysis. This group of DCIS lesions showed lower events of subsequent iIBC. Altogether, clinical H&E slides and data in model systems show that smaller DCIS duct sizes and non-spherical duct shapes are associated with a low progression rate.

A well curated dataset is key for successfully developing AI tools. The patient samples used in this study constitute one of the largest DCIS patient series treated with BCS only, long-term follow-up, well annotated clinical-pathological variables and with well-preserved tissue material (13). Moreover, we performed our analysis on a nested case-control study, based on a population-based DCIS cohort. As such, we have a strong enrichment for cases, in relation to other published hospital or cohort studies, in order to increase the power to find clinically relevant associations. A limitation of our study is that accurate external validation is very difficult to achieve, as large, well-curated, unbiased datasets, analogous to the dataset analyzed here on which AIDmap is based, are not available yet. Additionally, the accuracy of DCIS segmentation and detection in digital slides could still be optimized, as larger datasets come available.

Different approaches attempting to predict DCIS lesions according to risk of invasive progression have been proposed, including some using protein expression (11, 12, 13, 14), a panel of gene expression (16, 37), stromal expression patterns (27), immune cell composition (27, 38, 39) and clinical-pathological models (2, 10, 12, 15, 18, 19, 20). While interesting associations were reported, their potential to be translated to the clinic is unknown due to the lack of proper prediction accuracy tests. Our study addressed accuracy and probability of risk classification by applying penalized logistic regression and double-loop cross validation. The AUC values we got so far suggest that the morphometric signatures have clinical validity. In the case of molecular studies, the cost of such technologies could be an extra limitation. AIDmap is relatively low-cost, since it only requires inexpensive H&E slides, already routinely used in daily clinical practice.

Since AIDmap was developed based on tissue sections from BCS, it is essential to confirm its clinical utility in biopsies taken prior to treatment in guiding DCIS management. This is not trivial, because biopsies contain limited tissue that might not be fully representative of the DCIS lesion (20).

In conclusion, AIDmap is the first step on the road to a promising tool to identify women with indolent DCIS lesions and therefore may be spared surgery and/or radiotherapy, while not compromising the excellent outcomes of women with high-risk DCIS that do need treatment.

## Methods

### Study Population

We obtained data from a population-based cohort in which all women with primary DCIS without IBC between 1989 and 2004 in the Netherlands were included (26). From this cohort, H&E slides of FFPE tissue blocks, and well-annotated clinical and follow-up data were available (13, 26). Median follow-up time is 12 years (interquartile range 9.0–15.3 years). Based on this cohort, a nested case-control study including women treated with breast-conserving surgery (BCS) alone was designed. Patients diagnosed with iIBC during follow-up were considered as “cases” and those with no invasive diagnosis considered as “controls” (13). Matching between cases and controls was based on age at diagnosis and follow-up time. In addition, we obtained the expression levels of a series of molecular markers performed by immunohistochemistry, scored by pathologists, including ER, progesterone receptor (PR), HER2, COX-2, p53 and p16, as described previously (13). These samples have well-annotated morphological profile evaluated in previous studies (13, 15), such as histologic grade, necrosis, microcalcification, periductal fibrosis, periductal lymphocytes and DCIS growth patterns: a description of the proliferative architecture of DCIS defined as solid, cribriform, micropapillary, or clinging/flat epithelial atypia (FEA); all scored by pathologists, as previously described by Visser et al and Groen et al (13, 15).

### DCIS tissue and cell segmentation

Pathologists selected one H&E slide for each patient diagnosed with primary DCIS lesion, in which DCIS ducts were most representative in number and size. We scanned H&E whole-slide tissue sections using the scanner Pannoramic P1000 of 3D Histech at the Core Facility of Molecular Pathology and Biobanking (CFMPB) in the Netherlands Cancer Institute (NKI) with a 20x objective and a 0.24 microns/pixel resolution. We uploaded all WSIs in the HALO platform developed by IndicaLabs (https://indicalab.com/). First, 98 H&E slides were manually annotated to select the stroma area (figure 1). Next, pathologists digitally annotated DCIS regions in 54 H&E WSIs (supplementary appendix, section S1.1). Stroma and DCIS annotations were used by the HALO AI module, a deep learning neural network that created a trained-by-example tissue classifiers to detect stroma areas and DCIS ducts respectively. Additionally, we applied a nuclei segmentation classifier available in HALO to detect the nucleus of cells within the ducts. The HALO platform estimated the area, the perimeter and the spatial coordinates of these stroma areas, DCIS ducts and cell nucleus (figure 1). Finally, we applied a computational filtering using R studio to improve the detection accuracy (supplementary appendix section S1.2).

### Statistical Analyses

Tests and methods used in this study are detailed in the supplementary appendix (section S1.3). All statistical analyses were performed using R Studio version 4.2.2 (2022-10-31).

## Data Availability

Data collected and processed in this study is available to be shared upon request to the corresponding author.
